# Mutualism and Antagonism in a Post‐Extinction Hawaiian Bird–Lobelioid Pollination System

**DOI:** 10.1002/ece3.74123

**Published:** 2026-08-06

**Authors:** Samuel B. Case, Donald R. Drake, Kevin Epperly, Christopher Steinbronn, Keoki Kanakaokai, Molly E. Hagemann, Nathaniel H. Kingsley, Shannon Bay Gregory, Hanna L. Mounce, Alejandro Rico‐Guevara

**Affiliations:** ^1^ University of Washington Seattle WA USA; ^2^ Burke Museum of Natural History and Culture University of Washington Seattle WA USA; ^3^ Maui Forest Bird Recovery Project Makawao Hawaii USA; ^4^ Bernice Pauahi Bishop Museum Honolulu Hawaii USA; ^5^ University of Hawaiʻi at Mānoa Honolulu Hawaii USA; ^6^ Seed Conservation Laboratory, Harold L. Lyon Arboretum University of Hawaiʻi at Mānoa Honolulu Hawaii USA; ^7^ The Nature Conservancy, Hawaiʻi and Palmyra Honolulu Hawaii USA

**Keywords:** extinction, floral trait matching, Hawaiian lobelioids, nectar dynamics, nectar robbing, plant–animal interactions, pollination ecology, reproductive ecology

## Abstract

Extinction of specialized pollinators can alter the balance between mutualism and antagonism in plant–animal interactions, yet the mechanistic consequences of partner loss remain poorly resolved. We examined plant–bird interactions among three endangered Hawaiian lobelioids ( *Cyanea shipmanii* , *Clermontia pyrularia* , and *Clermontia lindseyana* ) and extant nectarivorous birds in montane forests on Hawaiʻi Island. These plants were likely pollinated historically by now‐extinct long‐billed Hawaiian honeycreepers, creating a system in which contemporary bird–plant interactions may differ from historical pollination dynamics. Using motion‐triggered cameras, we quantified floral visitation by ʻIʻiwi (*Drepanis coccinea*) and Hawaiʻi ʻAmakihi ( *Chlorodrepanis virens* ), evaluated whether nectar acquisition was coupled with contact with fertile floral structures, and measured the frequency and form of nectar robbing. To test the consequences of nectar robbing, we experimentally simulated corolla piercing and flower splitting using a 3D‐printed ʻAmakihi bill model, quantified subsequent nectar replenishment, and evaluated fruit development and seed viability. Contact with fertile floral structures (anthers or stigma) occurred in 43% of floral visits overall. Nectar robbing occurred in 39% of all visits and was more frequently performed by ʻAmakihi. Experimental damage significantly altered subsequent nectar replenishment, demonstrating that nectar robbing modifies floral resource dynamics beyond immediate removal. Despite frequent antagonistic behaviors and altered nectar dynamics, fruit production and seed viability remained high across treatments. These findings indicate that co‐occurring nectarivores perform different functional roles that alter floral resource dynamics and plant reproductive outcomes. In this post‐extinction system, mutualistic and antagonistic processes operate simultaneously, underscoring the importance of mechanistic evaluation when assessing the persistence of species interactions under biodiversity loss.

## Graphical abstract

Species loss can restructure plant–pollinator interactions, but the functional consequences of altered interactions remain unclear. Using camera observations and experimental manipulations in Hawaiian lobelioids, we show that nectarivorous birds differ in whether visits result in pollen contact or nectar robbing, with robbing altering nectar dynamics but not limiting plant reproductive success. These results demonstrate how alternative interaction pathways shape plant–animal interactions in communities affected by extinction.

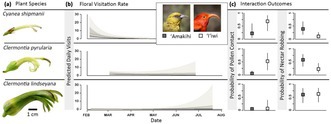

## 1 Introduction

Mutualistic interactions contribute to the persistence of species within ecological communities (Bascompte and Jordano 2007; Bronstein 2015). The stability of these interactions depends on the continued exchange of benefits between partners and on the behavioral and functional roles that species occupy within interaction networks (Bascompte and Jordano 2007; Thébault and Fontaine 2010). When extinctions remove interaction partners, remaining species may assume altered functional roles, shifting the balance between mutualistic and antagonistic interaction outcomes (Memmott et al. 2004; Valiente‐Banuet et al. 2015). Whether mutualistic function persists after partner loss depends on how remaining species interact and whether those interactions continue to generate reciprocal benefits.

In plant–pollinator mutualisms, flowering plants offer nectar and/or pollen rewards to animals that transfer pollen during feeding (Fenster et al. 2004; Ollerton et al. 2011). Coevolution between plants and pollinators can generate trait matching between floral and pollinator morphologies, increasing the efficiency and specificity of pollen transfer (Fenster et al. 2004; Temeles and Kress 2003; Rico‐Guevara et al. 2021). In bird‐pollinated systems, bill shape often corresponds to corolla length and curvature (Temeles and Kress 2003; Rico‐Guevara et al. 2019). Such trait associations are a hallmark of pollination syndromes and can promote efficient pollen transfer by aligning floral and pollinator morphology. Although trait matching between floral and pollinator morphology is well documented, the mechanistic consequences of this matching for interaction outcomes remain relatively understudied, particularly among less‐studied avian nectarivores (Hewes et al. 2025, 2022). When bills are shorter than floral tubes or otherwise misaligned with floral structure, birds may pierce or split flowers to access nectar, bypassing reproductive tissues (Traveset et al. 1998; Millikin et al. 2021; Colwell et al. 2023). In these nectar robbing events, interactions may shift toward antagonism and alter plant reproductive outcomes through both direct and indirect pathways (Irwin et al. 2010). Direct effects can arise from mechanical damage to floral structures, whereas indirect effects may occur through reduced nectar availability and changes in the attractiveness of flowers to subsequent pollinators. Because nectar is a shared resource among visitors, nectar robbing may therefore influence both the probability of pollen transfer and nectar availability for subsequent foraging by mutualists (or “legitimate” pollinators).

The Hawaiian Islands comprise a geographically isolated evolutionary system in which plant and bird lineages diversified over millions of years (Wagner and Funk 1995; Fleischer et al. 2008; Lerner et al. 2011). Endemic nectar‐feeding bird radiations included Hawaiian honeycreepers (Fringillidae) and the now‐extinct ʻŌʻō (Mohoidae) (Fleischer et al. 2008; Zhao et al. 2026), alongside the coevolved Hawaiian lobelioid radiation (Campanulaceae). For example, Hawaiian lobelioids exhibit long, curved tubular flowers characteristic of an ornithophilous pollination syndrome and consistent with adaptation to long‐billed avian pollinators, yet many are now pollination‐limited following the loss of these nectarivores (Case et al. 2026; Pender et al. 2014; Lammers and Freeman 1986). The ʻIʻiwi (*Drepanis coccinea*; syn. *Vestiaria coccinea*) now represents the longest‐billed extant nectarivore in many high‐elevation forests, where it forages alongside more generalist species such as the Hawaiʻi ʻAmakihi ( *Chlorodrepanis virens* , hereafter ʻAmakihi) (Fancy and Ralph 1998). Introduced birds also occupy remnant forests and interact with native flowers (Lammers et al. 1987; Aslan et al. 2014). Bird extinctions have substantially altered bill–flower trait matching across the Hawaiian Islands (Case et al. 2026), but the consequences of these changes for contemporary plant–bird interactions remain unresolved.

To address these uncertainties, we examined bird–plant interactions among endangered Hawaiian lobelioids and extant nectarivorous birds in high‐elevation forests on Hawaiʻi Island. We quantified floral visitation patterns by ʻIʻiwi and ʻAmakihi, evaluated whether nectar acquisition was coupled with pollen or stigma contact during individual floral visitations, and measured the frequency and form of nectar robbing. To assess whether antagonistic behaviors influence floral resources, we experimentally simulated damage caused by nectar robbing and quantified subsequent nectar replenishment across plant species. Finally, we evaluated fruit development and seed viability to determine whether altered interaction pathways translated into reduced reproductive output. We predicted that the shorter‐billed, more generalist ʻAmakihi would exhibit higher frequencies of nectar robbing and lower probabilities of pollen contact than ʻIʻiwi, particularly among plant species that differ in floral morphology and consequently in their degree of morphological matching with extant birds. We further predicted that nectar robbing would alter subsequent nectar dynamics and potentially reduce plant reproductive performance. By linking visitation behavior, pollen contact, nectar dynamics, and reproductive outcomes, this study evaluates the mechanisms through which contemporary bird–plant interactions influence floral resource dynamics and plant reproduction under a post‐extinction bird assemblage.

## 2 Methods

### 2.1 Study System

This study was conducted at Hakalau Forest National Wildlife Refuge on Mauna Kea, Hawaiʻi Island, USA. Fieldwork occurred in montane wet forest dominated by mature ʻōhiʻa lehua ( *Metrosideros polymorpha* ) and koa ( *Acacia koa* ) canopy at elevations of approximately 1400–2000 m. Hakalau supports one of the largest and most diverse remaining communities of native Hawaiian honeycreepers and functions as a stronghold for ʻIʻiwi, a species experiencing widespread declines at lower elevations across the archipelago due to avian malaria. Native nectarivores present include ʻIʻiwi, ʻAmakihi, and ʻApapane ( *Himatione sanguinea* ). Introduced species, including Warbling White‐eye ( *Zosterops japonicus* ) and Red‐billed Leiothrix ( *Leiothrix lutea* ), also occur in the refuge and were occasionally observed interacting with flowers. Focal plants were located within four ungulate‐free, fenced restoration outplantings established to augment declining native understory species, and plants occurred either as clustered groupings or as dispersed individuals. Although adult individuals persist and produce fruit within these exclosures, natural recruitment of lobelioids remains limited, consistent with broader patterns of regeneration failure across Hawaiian forests.

**FIGURE 1:**
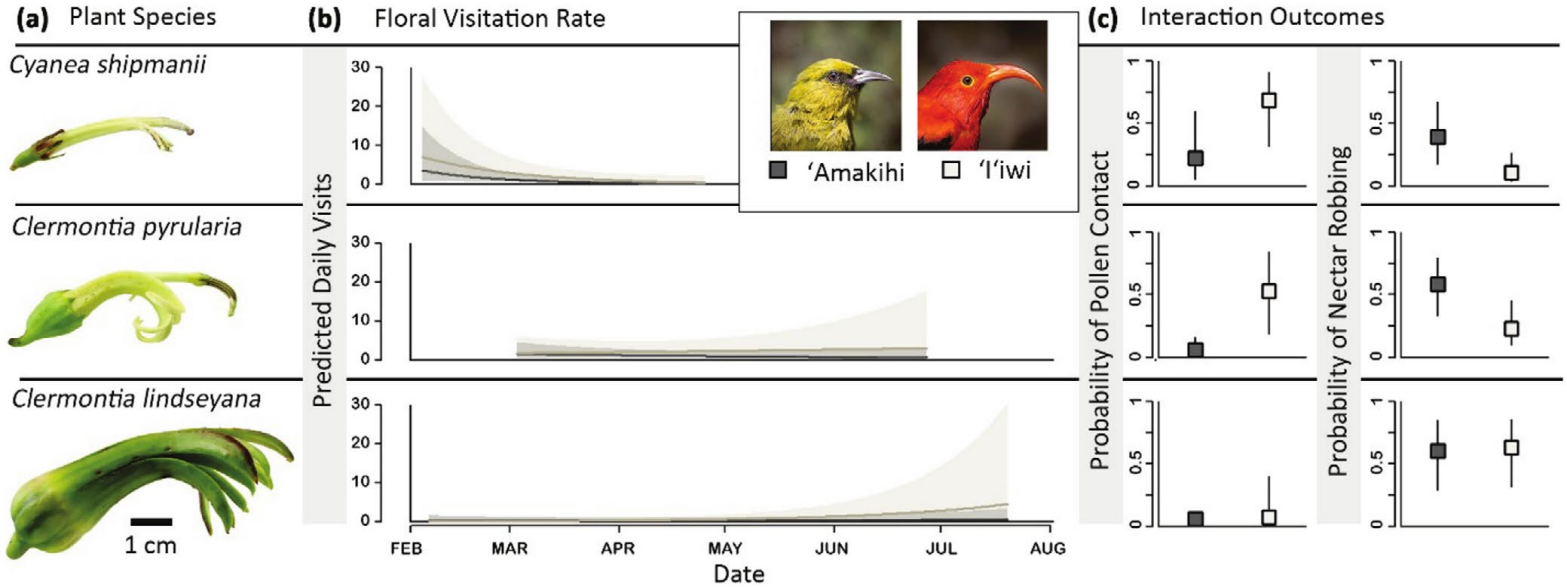
(a) Focal plant species examined: *Cyanea shipmanii* , *Clermontia pyrularia* , and *Clermontia lindseyana* . Photographs of flowers are shown to scale (scale bar = 1 cm). (b) Predicted daily floral visitation rate across the flowering season for each plant species, estimated from negative binomial generalized linear mixed models fit separately by plant species. Lines show model predictions for Hawaiʻi ʻAmakihi (dark gray) and ʻIʻiwi (light gray); shaded bands denote 95% confidence intervals. Predictions are shown only across the observed camera sampling window when open flowers were present. Bird photographs by Koa Matsuoka. (c) Predicted probabilities of pollen contact (left) and nectar robbing (right) during individual floral visitations, estimated from binomial generalized linear mixed models. Points show model‐predicted probabilities for each bird × plant combination; error bars denote 95% confidence intervals. Pollen contact and nectar robbing were assessed across a total of *n* = 1427 floral visits.

We focused on three Hawaiian lobelioid species: Hāhā ( *Cyanea shipmanii* ), and ʻŌhā wai ( *Clermontia pyrularia* and *Clermontia lindseyana* ). All three species are listed as endangered under the U.S. Endangered Species Act. Under IUCN Red List assessments, *C. lindseyana* is classified as Endangered, whereas *C. pyrularia* and *C. shipmanii* are classified as Critically Endangered. *C. pyrularia* and *C. shipmanii* are also designated as Plant Extinction Prevention Program (PEPP) species. These rare understory shrubs differ in floral morphology, growth form, and phenology (Wagner et al. 1999; Pender et al. 2014). Lobelioid flowers are tubular, bilaterally symmetrical, and protandrous, with nectaries located on top of the inferior ovary. Five petals form a fused corolla with distinct upper and lower lobes (Figure 1a). Stamens are united into a column surrounding the style, and pollen is released internally within this column. As the style elongates, it pushes pollen out of the tip of the staminal column, where it is presented on a brushy structure until the stigma emerges and the stigmatic lobes separate and become receptive, creating temporal separation of male and female function (Wagner et al. 1999; Pender et al. 2014). Corolla length, curvature, and orientation differ among species, traits likely influencing bill alignment, pollen contact, and nectar access (Figure 1a). Perianth structure also differs between the two *Clermontia* species. *C. pyrularia* possesses a perianth with a well‐developed corolla and reduced calyx, whereas *C. lindseyana* bears a well‐developed corolla surrounded by a calyx of five similarly sized sepals, producing a perianth with two whorls of similar length (Wagner et al. 1999). Flowering phenology varies by plant species and individual and occurs largely between January and July.

### 2.2 Camera Deployments

Motion‐activated cameras (Bushnell Core DS 4K No Glow Trail Camera) were deployed continuously during February–July 2025 on 34 randomly selected flowering individuals within outplanting areas, with one camera assigned to each focal plant. Cameras were positioned approximately 1.5–2.5 m from focal plants such that the maximum number of open flowers could be captured within the frame while maintaining comparable detection distance among deployments. Because flower number and plant architecture differed among lobelioid species, the number of open flowers visible within camera frames varied among deployments. Cameras were mounted on tripods staked into the ground unless suitable natural substrates were available for attachment and positioned to be as level with the flowers as possible while maintaining comparable detection distances and fields of view among plants.

Cameras were programmed to record 20‐s video clips per trigger using standard motion‐trigger settings. Cameras recorded continuously, including during nighttime hours. Deployments occurred only during budding or active flowering and remained in place until the end of the flowering stage. Cameras were checked approximately once per month to replace batteries and memory cards as needed. Cameras were deployed across four restoration outplantings, monitoring 34 flowering individuals and totaling 12,912 observation hours during periods when flowers were present.

### 2.3 Video Footage Processing

Video files were reviewed using a standardized protocol. For each camera deployment, the flowering window of the focal plant was defined by identifying the first and last videos containing open flowers, and only videos recorded within this interval were included in analyses. All videos recorded during the flowering window were viewed in full and floral visits were classified. A floral visit was defined as an animal physically interacting with a single flower. All animal visitors detected in footage, including birds and insects, were recorded; however, analyses were restricted to focal nectarivorous bird species (see Section 2.6). If a bird interacted with multiple flowers within the same video clip, each flower interaction was recorded as a separate floral visit. For each floral visit, we recorded bird species identity, nectar feeding, pollen contact, and nectar robbing, with nectar feeding defined as insertion of the bill into the flower and apparent consumption of nectar. Pollen contact was defined as any instance in which the bird's head or body contacted the pollen brush (male phase) or stigma (female phase).

Definitions of nectar robbing vary in the literature. Some authors restrict nectar robbing to nectar removal through perforations in floral tissue and distinguish nectar thieving as nectar removal through the floral opening without pollination (Inouye 1980; Maloof and Inouye 2000; Irwin et al. 2010). Given the complexity of floral interactions in our system and our interest in functional outcomes, we define nectar robbing operationally as nectar feeding during a floral visitation without contact with the pollen brush or stigma lobes. Under this definition, flower piercing, flower splitting, and morphological misalignment represent alternative behavioral pathways by which nectar is acquired without contact with fertile floral structures. Flower piercing involved puncturing the lateral wall of the fused perianth tube immediately above the ovary to access nectar. Flower splitting occurred when birds approached from behind the flower and split the dorsal perianth longitudinally from the floral opening downward toward the nectary, effectively opening the upper portion of the flower tube. Morphological misalignment occurred when nectar was accessed through the floral opening without observable contact with fertile floral structures.

### 2.4 Nectar Robbing Damage Experiment

To evaluate the effects of nectar robbing on nectar replenishment, we experimentally simulated two forms of damage observed during floral visitations: flower piercing and flower splitting. Piercing damage was applied using a 3D model of an ʻAmakihi bill constructed to replicate the observed nectar‐robbing behaviors. A Hawaiʻi ʻAmakihi museum specimen was scanned using photogrammetry, reconstructed in Agisoft Metashape, and converted into a digital model replicating bill curvature and tip morphology (detailed methods in Medina et al. 2024 and Garzón‐Agudelo et al. 2025). The model was printed using rigid polymer filament (PLA) and used to puncture the floral tube in a manner consistent with piercing behaviors documented in our video footage. Splitting damage was replicated by inserting the bill into the open mouth of the flower and sliding it downward along the dorsal sutures joining the perianth lobes, opening the upper portion of the floral tube as observed during previously filmed feeding events.

Experimental plants were selected within adjacent fenced outplantings located in the same area of the refuge to minimize environmental variation. All flowers were bagged in bud to prevent natural visitation before the experiment began. Open flowers were sampled between 8:00 and 12:00 on Day 1, on April 15, 2025. All nectar present at the time of sampling was completely removed from flowers prior to treatment to standardize starting nectar volumes across treatments and ensure that subsequent measurements reflected nectar replenishment occurring after experimental damage. Nectar volume was measured using 20 μL glass microcapillary tubes; nectar column length was measured with digital calipers and converted to volume based on tube calibration (6.4 cm = 20 μL) to establish pre‐treatment baseline volume. When nectar volume exceeded the capacity of a single tube, nectar was drawn into multiple tubes of the same size and column lengths were summed prior to conversion. Nectar sugar concentration (% sucrose equivalents) was measured using microcapillary tubes and a handheld refractometer. Flowers were then randomly assigned to one of three treatments: control (no damage), piercing, or splitting. Control flowers were handled identically to damaged flowers but received no experimental manipulation (besides nectar extraction). Because lobelioid flowers are protandrous (Devlin and Stephenson 1985), floral sex stage (male or female) was recorded at the time of treatment and included as a proxy for floral age.

Across species, treatment sample sizes were as follows: *C. lindseyana* (control = 12, piercing = 10, splitting = 9), *C. pyrularia* (control = 16, piercing = 9, splitting = 8), and *C. shipmanii* (control = 17, piercing = 15, splitting = 12). Sample sizes varied among species due to phenological differences and conservation constraints associated with working on federally endangered plants. A total of 54 male‐stage and 54 female‐stage flowers were included.

Following treatment, flowers remained bagged except during nectar measurements. Nectar volume and sugar concentration were re‐measured after 24 h. After Day 2 measurements, flowers remained bagged and were monitored for fruit development. Fruit set, including whether fruits matured or aborted, was assessed 21 days later.

### 2.5 Seed Viability and Germination Analyses

Following assessment of fruit development in experimentally treated flowers, mature fruits were collected from a subset of experimental plants for seed viability and germination analyses (final sample sizes reported in Section 3). Because fruit ripening did not occur synchronously across species and treatments, only fruits that reached maturity during the collection window were harvested for seed analysis. A small number of fruits from unbagged flowers were collected to confirm seed viability under natural pollination conditions (see Section 3 for sample sizes). Fruits were collected under U.S. Fish and Wildlife Service Recovery Permit No. PER14302826 and Refuge Special Use Permit 12516‐25001‐R.

Seeds were manually extracted from fruit pulp, cleaned, and air‐dried at approximately 20°C prior to analysis. All submitted seeds were subjected to X‐ray imaging to assess seed fill status. Seeds were scored as filled based on visible embryo development and as unfilled when seeds appeared empty, damaged, or lacked visible internal tissue. Seed fill was used as a non‐destructive proxy for potential seed viability and germination potential (Liu et al. 2020), and seed counts per collection were recorded. For *C. shipmanii* , two collections were additionally subjected to germination trials under standard laboratory conditions to validate the X‐ray assessment of seed fill. Germination assays followed general seed‐testing principles for substrate, hydration solution, temperature, and pre‐treatments to maximize germination proportion and minimize assay duration (AOSA 2018). Because published germination protocols were unavailable for the focal taxa, germination conditions were also guided by institutional experience, information on dormancy mechanisms and dormancy‐breaking treatments for congeners (Baskin and Baskin 2014), and climate data during seed maturation and seedling emergence periods.

Twenty‐five seeds from each collection were sown on 1% agar and incubated in a Percival E36VL growth chamber. No dormancy‐breaking pre‐treatments or scarification procedures were applied, as institutional experience indicates that seeds of Hawaiian lobelioids exhibit no additional germination requirements beyond adequate moisture and warmth. Diurnal conditions in the growth chamber ranged from 22°C–23°C (day) to 16°C–17°C (night), with relative humidity maintained at > 60%. Photoperiods were adjusted monthly to approximate local seasonal conditions, with 11–12 h of light per day delivered during the higher‐temperature periods. Seeds were scored weekly, and germination was recorded when the radicle length reached ≥ 2 mm. Tests concluded when no new germination occurred for at least 8 weeks or when all seeds had germinated or had lost integrity (softened or become moldy). Because seed fill and germination rates were uniformly high across species and treatments, seed data were summarized descriptively and were not included in statistical models.

### 2.6 Statistical Analyses

We used generalized linear mixed models to account for non‐independence among observations at the plant and outplanting levels. Analyses focused on ʻIʻiwi and ʻAmakihi because these species accounted for 96% of all recorded floral visits (Table 1), whereas other visitors each contributed fewer than 20 visitation events across the study. Rare visitors included Warbling White‐eye, Red‐billed Leiothrix, and introduced honey bees, whose low frequencies precluded species‐level analysis and were unlikely to influence overall interaction patterns or floral damage. Records with ambiguous bird identity were excluded from species‐level analyses, although unidentified visits were retained in overall visitation totals reported in Table 1. No rodent visitation events were detected during the study period. Direct pollen deposition could not be measured from video footage; therefore, pollen contact should be interpreted as a proxy for potential pollen transfer rather than a direct measure of pollination success. All analyses were conducted in R version 4.5.2 (R Core Team 2024). Models were implemented using the glmmTMB and lme4 packages, and model diagnostics were evaluated using the DHARMa package (Brooks et al. 2017; Bates et al. 2015; Hartig 2022).

**TABLE 1:** Floral visitation events recorded by motion‐triggered cameras across three lobelioid species.

Visitor	*Clermontia lindseyana*	*Clermontia pyrularia*	*Cyanea shipmanii*	Total
ʻIʻiwi	275	648	453	1376
ʻAmakihi	94	303	176	573
Warbling White‐eye	3	1	0	4
Red‐billed Leiothrix	1	0	18	19
Hymenoptera	0	1	29	30
Unidentified	12	19	5	36
Total	373	953	676	2038

*Note:* Counts represent individual floral visitation events extracted from motion‐triggered camera footage. Totals include all detected visitation events during the study period prior to taxonomic filtering for focal‐species analyses. Hymenoptera records represent insect visitors identified to order level only; detection of these visitors may be underrepresented due to the motion‐triggered detection mechanism of the cameras. “Unidentified” records represent bird visitation events for which species identity could not be reliably determined due to camera angle, partial obstruction, bird positioning within the frame, or lighting limitations.

#### 2.6.1 Floral Visitation

To evaluate temporal patterns in bird visitation, we modeled daily rates of floral visitation. Individual floral visits were aggregated to daily counts for each bird species on each plant. Analyses were restricted to days when flowers were present and open, and camera detection was possible. Aggregation to the daily level provided a consistent sampling unit and allowed retention of biologically meaningful zeroes. Because the number of open flowers visible within camera frames varied among deployments and was not recorded daily, visitation rates represent plant‐level activity rather than per‐flower visitation rates, with differences in typical floral display size among species partially accounted for by inclusion of plant species as a fixed effect.

Separate models were fit for each lobelioid species. The response variable was the daily count of floral visits. Daily visitation counts were modeled with a negative binomial error distribution. Fixed effects included bird species, Julian date, and their interaction. Random intercepts were included for plant identity nested within outplanting identity. Individual visitation events were aggregated prior to analysis, such that the sampling unit for these models was the plant × day × bird species combination.

#### 2.6.2 Interaction Outcomes

Separate models were fit for pollen contact and nectar robbing, with each response variable scored as a binary outcome (1 = yes, 0 = no). Analyses were conducted at the level of individual flower interactions. Records were filtered to include only focal bird species and the three focal lobelioid species. Sample sizes differed slightly between analyses because nectar robbing behavior could not be confidently scored in a small subset of visits due to incomplete visibility of feeding position.

Both outcomes were analyzed using binomial error distributions with logit link functions. Fixed effects included bird species, plant species, and their interaction. Random intercepts were included for plant identity nested within outplanting.

#### 2.6.3 Nectar Replenishment Experiment

To evaluate treatment effects on nectar replenishment, proportional change in nectar volume was calculated as a log response ratio:

$$\log\left(\frac{V_{2}+\varepsilon }{V_{1}+\varepsilon }\right)$$

where 𝑉₁ and 𝑉₂ represent nectar volume on Day 1 and Day 2, respectively, and 𝜀 was defined as half of the smallest non‐zero nectar measurement observed in the dataset (𝜀 = 0.5 μL). Adding this constant allowed symmetric interpretation of increases and decreases in nectar volume while avoiding undefined values associated with zeros (Hedges et al. 1999). The resulting metric quantifies replenishment relative to initial nectar volume, enabling comparisons among flowers that differed substantially in starting nectar amounts and isolating treatment effects on replenishment dynamics rather than absolute nectar availability. Because proportional change was calculated relative to each flower's initial nectar volume, treatment comparisons were independent of baseline differences in nectar standing crop among flowers.

The response variable was the log response ratio describing proportional change in nectar volume over the 24‐h sampling interval. Log response ratios were analyzed using linear mixed‐effects models implemented in the lme4 package. Fixed effects included plant species, treatment (control, piercing damage, or splitting damage), their interaction, and floral sex stage (male vs. female), which served as a proxy for flower age due to protandry. A random intercept for plant identity was included to account for repeated sampling of flowers from the same plant. Sugar concentration data were summarized descriptively (Table S1) but were not modeled statistically because repeated nectar extraction and the 24‐h sampling interval were not designed to isolate treatment effects on nectar chemistry.

## 3 Results

### 3.1 Summary of Observations

Across three focal lobelioid species (*C. shipmanii*, *C. pyrularia*, *C. lindseyana*), we monitored 34 flowering individuals across four outplantings, totaling 12,912 observation hours during periods when flowers were present. Across all footage, we recorded 2038 floral visitation events (Table 1), of which 1949 involved focal native nectarivores (ʻAmakihi and ʻIʻiwi). From these interactions, we retained 1427 visits for which pollen contact could be confidently determined. When contact occurred (*n* = 650), it involved the head in 90% of cases and the body in 13.2% (not mutually exclusive). Non‐native birds were observed visiting flowers on 23 occasions, and pollen contact was detected in one non‐native bird visit. Bees were detected on 30 occasions; observed behavior consisted primarily of pollen collection, though fine‐scale pollen deposition could not be assessed due to camera resolution constraints.

### 3.2 Seasonal Patterns of Floral Visitation

Daily visitation rates varied across the flowering season, influencing opportunities for contact with fertile floral structures and nectar robbing (Figure 1b; Table 2). For *C. shipmanii*, visitation declined significantly with increasing Julian date, indicating higher early‐season visitation followed by reduced visitation later in the season. Seasonal trends for *C. pyrularia* were weaker and not statistically significant, with substantial overlap in visitation rates across the flowering window. For *C. lindseyana*, overall visitation rates differed between bird species, with ʻIʻiwi exhibiting significantly lower visitation early in the season relative to ʻAmakihi. However, a significant bird × date interaction indicated that ʻIʻiwi visitation increased relative to ʻAmakihi as the season progressed, resulting in convergence of visitation rates later in the season. Across plant species, seasonal patterns of visitation were broadly similar between bird species for *C. shipmanii* and *C. pyrularia*, whereas *C. lindseyana* exhibited evidence of species‐specific temporal dynamics. These patterns may also reflect staggered flowering dynamics among co‐occurring lobelioids, potentially redistributing visitation across species through the season, a possibility consistent with temporal variation in floral availability.

**TABLE 2:** Seasonal patterns of floral visitation to three lobelioid species.

Effect	Estimate	SE	*z*	*p*
*Cyanea shipmanii*				
Intercept	2.777	1.308	2.12	0.034
Bird (ʻIʻiwi)	0.413	1.552	0.27	0.790
Julian date	−0.044	0.018	−2.45	0.014
Bird × Julian date	0.008	0.020	0.41	0.684
*Clermontia pyrularia*				
Intercept	0.901	1.048	0.86	0.390
Bird (ʻIʻiwi)	−0.568	0.998	−0.57	0.569
Julian date	−0.007	0.009	−0.79	0.431
Bird × Julian date	0.012	0.008	1.40	0.162
*Clermontia lindseyana*				
Intercept	−1.335	1.143	−1.17	0.243
Bird (ʻIʻiwi)	−2.076	0.900	−2.31	0.021
Julian date	0.003	0.009	0.30	0.765
Bird × Julian date	0.022	0.006	3.46	< 0.001

*Note:* Negative binomial generalized linear mixed models (log link) of daily floral visitation counts fitted separately for each plant species. Fixed effects included bird species, Julian date, and their interaction; plant ID nested within outplanting was included as a random intercept. ʻAmakihi is the reference bird species. Estimates are on the log scale. Sample sizes correspond to daily plant × bird observations for each species.

### 3.3 Floral Interaction Outcomes

#### 3.3.1 Pollen Contact

Of the 1949 focal bird visits recorded, 1427 interactions could be confidently scored for pollen contact and were retained for analysis. Pollen contact occurred in 43% of these visits. Baseline pollen contact probability (for ʻAmakihi on *C. lindseyana*) was the lowest among all bird–plant combinations (Table 3, Figure 1c). ʻIʻiwi were significantly more likely to contact pollen than ʻAmakihi on *C. lindseyana* (logit = 2.15 ± 1.01 SE, *p* = 0.033), with odds of pollen contact more than eight times higher. Plant species also influenced pollen contact: relative to *C. lindseyana*, *C. shipmanii* exhibited significantly higher pollen contact probability (logit = 3.53 ± 1.54 SE, *p* = 0.022), whereas *C. pyrularia* did not differ significantly. Interaction terms between bird and plant species were not significant, indicating that the difference in pollen contact probability between ʻIʻiwi and ʻAmakihi was consistent across plant species.

**TABLE 3:** Effects of bird species and plant species on floral interaction outcomes.

Effect	Estimate	SE	*z*	*p*
A. Pollen contact model				
Intercept (ʻAmakihi, *C. lindseyana*)	−4.765	1.311	−3.63	< 0.001
Bird (ʻIʻiwi)	2.145	1.009	2.13	0.033
Plant (*C. pyrularia*)	1.488	1.547	0.96	0.336
Plant (*C. shipmanii*)	3.529	1.545	2.28	0.022
ʻIʻiwi × *C. pyrularia*	1.251	1.084	1.15	0.249
ʻIʻiwi × *C. shipmanii*	−0.113	1.119	−0.10	0.919
B. Nectar robbing model				
Intercept (ʻAmakihi, *C. lindseyana*)	0.424	0.672	0.63	0.528
Bird (ʻIʻiwi)	0.108	0.442	0.25	0.806
Plant (*C. pyrularia*)	−0.090	0.774	−0.12	0.907
Plant (*C. shipmanii*)	−0.848	0.765	−1.11	0.268
ʻIʻiwi × *C. pyrularia*	−1.649	0.497	−3.32	< 0.001
ʻIʻiwi × *C. shipmanii*	−1.807	0.631	−2.87	0.004

*Note:* Binomial generalized linear mixed models (logit link) of (A) pollen contact and (B) nectar robbing during individual floral visits. Fixed effects included bird species, plant species, and their interaction; plant ID nested within outplanting was included as a random intercept. ʻAmakihi and *C. lindseyana* are reference levels. Estimates are on the logit scale.

#### 3.3.2 Nectar Robbing

Across the 1427 floral visits analyzed, nectar robbing occurred in 38% of observations. For *C. lindseyana*, nectar robbing probability did not differ between ʻIʻiwi and ʻAmakihi (*p* = 0.81) (Table 3, Figure 1c). However, significant bird × plant interactions indicated plant‐specific differences in robbing behavior. On *C. pyrularia*, ʻIʻiwi exhibited significantly lower nectar robbing probability relative to ʻAmakihi (interaction logit = −1.65 ± 0.50 SE, *p* < 0.001); ʻIʻiwi had approximately 81% lower odds of robbing compared to ʻAmakihi on this species. Similarly, on *C. shipmanii*, ʻIʻiwi showed significantly reduced robbing probability relative to ʻAmakihi (interaction logit = −1.81 ± 0.63 SE, *p* = 0.004), with an approximately 84% reduction in robbing odds. Thus, nectar‐robbing behavior differed between bird species in a plant‐dependent manner, whereas pollen contact differences were more consistent across plant taxa. Notably, *C. lindseyana* combined the lowest observed pollen contact probabilities with relatively high nectar robbing frequency compared to the other lobelioid species.

### 3.4 Nectar Replenishment Experiment

**FIGURE 2:**
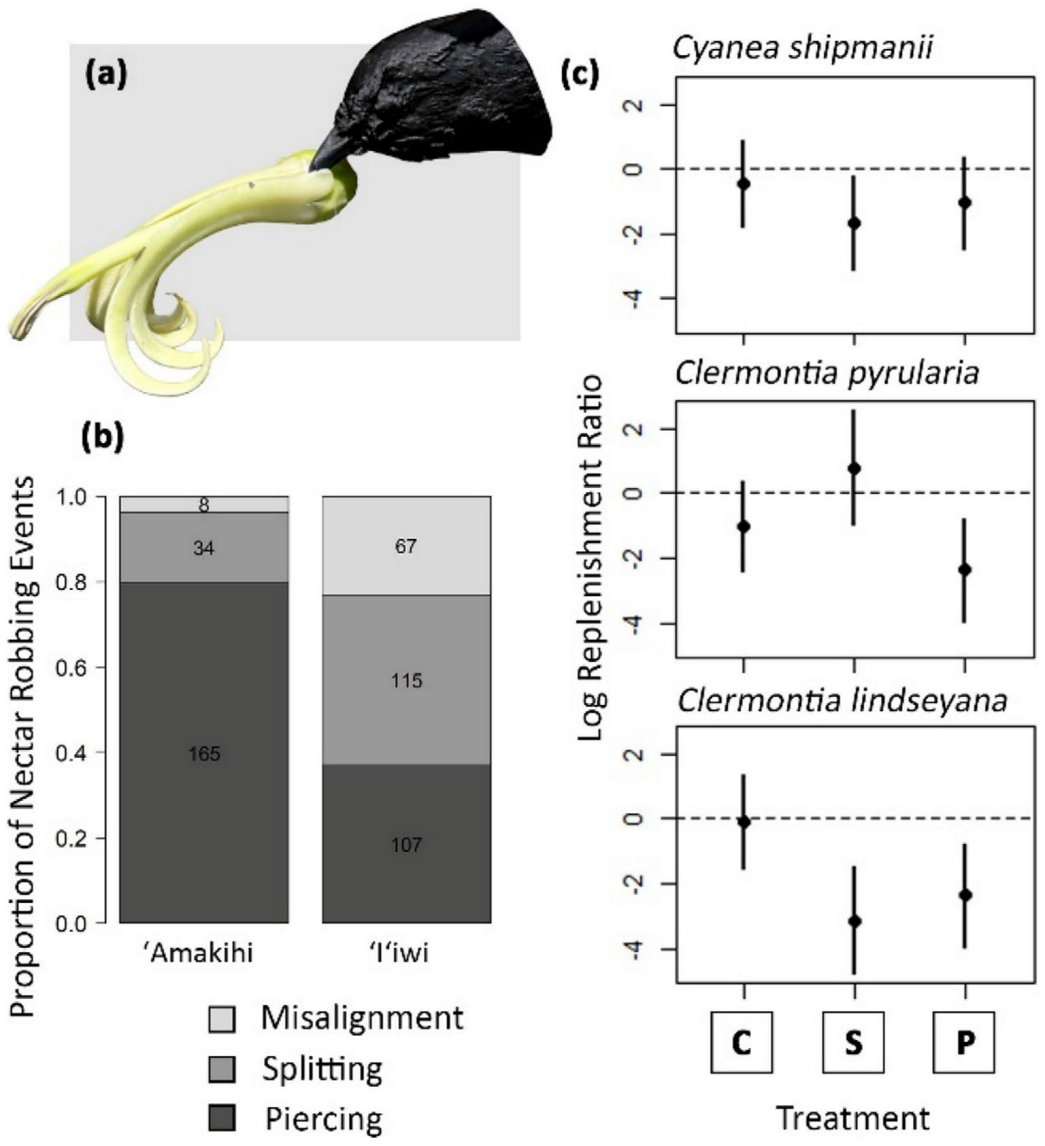
(a) Experimental simulation of nectar robbing damage using a 3D‐printed Hawaiʻi ʻAmakihi bill model. Photograph shows the flower splitting treatment applied to a *Clermontia pyrularia* flower. (b) Composition of nectar robbing behaviors observed in camera footage, expressed as the proportion of nectar robbing events classified as floral piercing, flower splitting, or apparent morphological misalignment (nectar acquisition without observable contact with fertile floral structures). Numbers within bars indicate the observed count of nectar robbing events in each category: ʻAmakihi (piercing = 165, splitting = 34, misalignment = 8) and ʻIʻiwi (piercing = 107, splitting = 115, misalignment = 67). (c) Effects of simulated nectar robbing damage on nectar replenishment for each plant species. Points show model‐estimated mean log response ratios of nectar volume between Day 2 and Day 1; error bars denote 95% confidence intervals. Values above zero indicate increased nectar volume between sampling days, whereas values below zero indicate reduced replenishment. Treatments are C (control, no damage), S (flower splitting), and P (flower piercing). All flowers were bagged prior to anthesis and remained bagged throughout the experiment to exclude natural visitation (*n* = 108 flowers from 27 plants).

We used a 3D‐printed ʻAmakihi bill replica to experimentally simulate flower piercing and flower splitting observed during nectar robbing events (Figure 2a). Although the proportional use of splitting and piercing differed between ʻIʻiwi and ʻAmakihi, both species employed both nectar robbing behaviors to access nectar, indicating behavioral flexibility in nectar extraction (Figure 2b).

Across 108 experimental flowers from 27 plants, nectar replenishment responses varied significantly among treatments and plant species (Table 4, Figure 2c). The relative effects of damage from piercing and splitting differed among plant species, indicating species‐dependent responses to simulated nectar robbing. For *C. lindseyana* (the model reference category), simulated piercing and splitting both substantially reduced nectar replenishment relative to control flowers. Piercing reduced replenishment to approximately 10% of control levels (log response ratio = −2.27), whereas splitting reduced replenishment to approximately 5% of control levels (log response ratio = −3.04). Treatment effects differed among plant species. For *C. pyrularia*, the effect of splitting differed significantly from *C. lindseyana* (interaction term = 4.85), resulting in a net positive replenishment response relative to control; splitting increased nectar replenishment to approximately six times control levels in *C. pyrularia*. In contrast, piercing in *C. pyrularia* reduced replenishment to approximately 26% of control levels. For *C. shipmanii*, both damage types reduced nectar replenishment relative to controls, with splitting producing a stronger reduction than piercing; however, the magnitude of reduction was smaller than in *C. lindseyana*. Piercing reduced replenishment to approximately 55% of control levels, whereas splitting reduced replenishment to approximately 30% of control levels. Floral sex stage influenced nectar replenishment independently of treatment. Male‐stage flowers exhibited approximately twofold higher replenishment rates than female‐stage flowers (log response ratio = 0.71), although this effect was moderate relative to treatment and species effects (Table 4). Substantial among‐plant variation was detected (plant‐level standard deviation = 0.83), indicating heterogeneity in nectar replenishment capacity among individual plants (Table 4). Nectar sugar concentration generally declined from Day 1 to Day 2 across plant species and treatments, including control flowers (Table S1), suggesting that temporal dynamics following nectar removal may contribute to observed variation. Following simulated nectar robbing damage, three fruits aborted (two *C. pyrularia* and one *C. lindseyana*); however, in all other cases, fruits remained attached and continued maturation. Five flowers of *C. lindseyana* (16%), nine of *C. pyrularia* (27%), and nineteen of *C. shipmanii* (43%) contained no measurable nectar at baseline despite being bagged prior to anthesis.

**TABLE 4:** Effects of simulated nectar robbing damage on nectar replenishment.

Effect	Estimate	SE	*t*
Intercept	−0.087	0.747	−0.12
Plant species: *C. pyrularia*	−0.939	0.979	−0.96
Plant species: *C. shipmanii*	−0.357	0.973	−0.37
Treatment: Piercing	−2.274	0.982	−2.31
Treatment: Splitting	−3.045	1.010	−3.01
Floral sex stage: Male	0.709	0.480	1.48
C. pyrularia × Piercing	0.933	1.379	0.68
*C. shipmanii* × Piercing	1.681	1.265	1.33
*C. pyrularia* × Splitting	4.849	1.397	3.47
*C. shipmanii* × Splitting	1.822	1.321	1.38

*Note:* Linear mixed‐effects model of log nectar replenishment ratio between Day 1 and Day 2 volumes. Fixed effects included plant species, treatment (control, piercing, splitting), their interaction, and floral sex stage; plant ID was included as a random intercept. *C. lindseyana*, control treatment (C), and the female floral sex stage serve as reference levels. Estimates are on the log response ratio scale.

### 3.5 Seed Viability and Germination

Mature fruits from the nectar‐replenishment experiment were collected from *C. shipmanii* (*n* = 30 fruits, *n* = 4263 seeds). To compare with baseline seed set under natural exposure, we additionally collected unbagged fruits including 53 *C. shipmanii* (*n* = 9663 seeds), 15 *C. pyrularia* (*n* = 472 seeds), and 20 *C. lindseyana* fruits (*n* = 4725 seeds). X‐ray imaging indicated that the vast majority of seeds were filled across species and collections. For *C. lindseyana* and *C. pyrularia*, all seeds were filled except for one empty seed detected in *C. pyrularia*. Overall, seed fill exceeded 99% across collections. High seed fill was observed in both experimentally bagged flowers and the small number of naturally exposed fruits collected for comparison. For *C. shipmanii*, germination trials were conducted on a subset of seeds from two collections (*n* = 25 seeds per collection), each of which yielded 96% germination. No qualitative differences in seed fill or germination were observed among nectar robbing treatments or between bagged and unbagged flowers.

## 4 Discussion

Widespread extinction of specialized nectarivores in Hawaiʻi has disrupted coevolved bird–plant mutualisms, raising uncertainty about the mechanisms through which remaining interactions function as pollination mutualisms or shift toward antagonism. In this system, ʻIʻiwi and ʻAmakihi overlapped extensively in floral visitation across all three lobelioid species examined, indicating a shared use of floral resources despite simplified pollinator assemblages. However, their functional roles differed: ʻIʻiwi were substantially more likely to contact pollen during floral visits, whereas ʻAmakihi exhibited higher frequencies of nectar robbing on two plant species. Both species engaged in flower piercing and flower splitting, behaviors that varied among plant species and may be associated with differences in the degree of morphological matching between flowers and bills (Tables S2 and S3). Notably, on the species with the longest corollas (*C. lindseyana*), both birds exhibited low pollen contact and high robbing frequencies, consistent with reduced morphological matching relative to the other lobelioids. Experimental simulation of these robbing behaviors demonstrated that nectar removal via floral damage can alter subsequent nectar replenishment in a plant‐specific manner, with strong reductions in *C. lindseyana*, moderate reductions in *C. shipmanii*, and increased replenishment following the splitting method in *C. pyrularia*. These results indicate that antagonistic behaviors are common and can modify floral resource dynamics, potentially influencing subsequent foraging interactions. Nevertheless, we found no evidence that these behavioral differences translated into reduced seed viability in sampled fruits. Together, these findings indicate that extinction‐driven changes in pollinator composition may have reshaped interaction pathways and are consistent with increased opportunities for antagonistic interactions, yet opportunities for pollen transfer and successful seed production persist under the current bird assemblage. These patterns likely reflect interaction dynamics that have emerged following the loss of specialized nectarivores and the simplification of Hawaiian pollination networks.

### 4.1 Post‐Extinction Pollination Networks in Hawaiʻi

The interaction network examined here reflects a post‐extinction ecosystem (Case and Tarwater 2020; Soares et al. 2022). Historical accounts indicate that long‐billed nectar specialists such as the Hawaiʻi Mamo (*Drepanis pacifica*) or Hawaiʻi ʻŌʻō (*Moho nobilis*) once occupied these forests and defended lobelioid nectar resources (Perkins 1903; Olson and James 1982). Lobelioid communities were also much more diverse and abundant in the understory prior to disturbance by introduced ungulates and habitat modification (Rock 1913; Wagner et al. 1999). The loss of these nectarivore and lobelioid species, together with reduced lobelioid recruitment in the present system, has altered the structure of plant–bird interactions in the Hawaiian Islands (Cox 1983). Following the loss of specialists, remaining nectarivores now share floral resources that were once partitioned among a more functionally diverse assemblage (Sayol et al. 2021). Such changes likely shift interaction gradients between mutualism and antagonism, particularly when trait matching between birds and flowers is reduced (Case et al. 2026).

### 4.2 Resource Dynamics and the Consequences of Nectar Robbing

Unlike typical nectar removal, in which depletion reduces resource availability only until nectar is replenished, nectar robbing in this system altered subsequent nectar production. Experimental damage to flowers reduced nectar replenishment in *C. lindseyana* and *C. shipmanii* and, in some cases, effectively halted nectar production. In *C. pyrularia*, splitting damage increased nectar replenishment, demonstrating that the consequences of floral damage are specific to species and robbing method. The strong negative response observed in *C. lindseyana* may reflect differences in perianth structure, as this species possesses two perianth whorls and thus requires greater damage to access nectar. These results indicate that nectar robbing can modify the resource landscape beyond immediate depletion, potentially influencing subsequent visitation patterns and competitive dynamics among birds. More broadly, nectar robbing can impose physiological and fitness costs on plants through direct damage to floral tissues, altered pollinator behavior, and reductions in plant reproductive success, although the magnitude and direction of these effects vary considerably among systems (Irwin et al. 2010). Our results suggest an additional pathway by which robbing may influence plant fitness by altering subsequent nectar production and resource availability, potentially modifying future interactions between plants and floral visitors. Descriptive sucrose measurements suggested that nectar concentration often declined over the 24‐h replenishment interval even in control flowers (Table S1), consistent with the possibility that nectar volume replenishment and sugar accumulation occur on different timescales; targeted sampling designs will be needed to test effects of robbing on nectar quality.

We also observed that some flowers bagged in bud contained no measurable nectar at baseline. Among flowers lacking measurable nectar at baseline across species (*n* = 33), nine subsequently produced nectar by the following measurement period, indicating that nectar secretion can initiate after an initial absence. Because these flowers had not yet been naturally visited, nectar production may be temporally variable or responsive to mechanical disturbance associated with experimental manipulation, consistent with documented variability in nectar secretion among flowering plants (Willmer 2011). One possibility is that floral stimulation during visitation could influence nectar secretion dynamics, although this mechanism was not directly tested here. The ecological significance of such variation remains unclear and warrants further investigation into temporal patterns of nectar availability.

### 4.3 Behavioral Interactions and Competitive Structure

Field observations revealed social interactions among birds at focal plants, including chasing and displacement within and between species. These behaviors suggest that both exploitative and interference competition may structure nectar access (Rico‐Guevara et al. 2021; Sargent et al. 2021). Distinguishing the relative importance of resource depletion versus behavioral exclusion will require further study. Seasonal overlap in visitation and similar overall visitation rates among plant species indicate that birds may compensate for smaller floral rewards by increasing the number of flowers visited per plant, particularly in species such as *C. shipmanii* that bear smaller but more numerous flowers. Nectar standing crop and sugar composition have been described for some Hawaiian lobelioids (Pender et al. 2014), yet quantitative data on nectar production rates and temporal variability remain limited. Improved understanding of how floral rewards vary through time and respond to visitation could help clarify how behavioral interactions among nectarivores translate into competitive dynamics at flowers.

### 4.4 Trait Matching and Plant‐Specific Responses

Plant species differed in floral morphology (Tables S2 and S3), and descriptive comparisons of bill and floral dimensions suggest decreasing proportional trait matching with increasing flower length. Consistent with this pattern, larger flowers were less likely to receive pollen contact and more likely to be nectar robbed, consistent with observations from other bird‐pollinated systems in which morphological matching influences interaction outcomes (Temeles and Kress 2003; Irwin et al. 2010; Fenster et al. 2004). Flowers also differed in their responses to experimental nectar robbing damage. *C. lindseyana*, which has the largest floral tube, exhibited the strongest reductions in nectar replenishment following damage. Visitation rates were not markedly different among plant species, suggesting that larger flowers remained attractive to birds despite exhibiting lower probabilities of pollen contact and higher frequencies of nectar robbing. Together, these results are more consistent with incomplete morphological matching than with reduced floral attractiveness. Imperfect trait matching therefore does not appear to prevent visitation but may instead shift the functional outcome of interactions between pollination and nectar robbing. The predominance of pollen contact on the head during feeding further highlights the importance of functional alignment between bird morphology and floral structure. The importance of functional alignment may be particularly pronounced in Hawaiʻi, where extinction has removed much of the historical diversity of long‐billed nectar specialists and altered the functional composition of pollinator assemblages (Case et al. 2026).

### 4.5 Conservation Implications and Future Directions

The Hawaiian lobelioid radiation is of immediate conservation concern, with 35% of species currently threatened or endangered and 21% considered extinct (Rose et al. 2025). The present bird–lobelioid network does not reflect the historical configuration under which these species evolved (Perkins 1903; Olson and James 1982; Wagner et al. 1999; Pratt et al. 2009). Loss of specialized nectarivores, incomplete trait matching, and seasonal limitations in floral availability suggest that mutualistic function may not be optimized under current conditions (Case et al. 2026; Matthews et al. 2024). At the same time, fruit production persisted following experimental floral damage, and seed viability remained high, indicating resilience in reproductive output. We found that both experimentally bagged flowers and a small set of naturally exposed (unbagged) flowers produced ripe fruits with viable seeds, suggesting the capacity for self‐fertilization may be a critical adaptation buffering lobelioids against pollinator loss. However, reliance on self‐fertilization may carry long‐term costs, as animal‐mediated pollination promotes genetic diversity, gene flow among populations, and adaptive potential in fragmented landscapes. The extent to which contemporary interactions maintain pollen movement, gene flow, and outcrossing rates remains unknown and warrants further investigation.

Invasive grasses in the understory and pressure from introduced animals (Cole et al. 2012) limit native plant recruitment at Hakalau. Conservation efforts should prioritize understanding recruitment limitations and expanding out‐plantings of lobelioids and other native flowering species. Restoring floral resource diversity, including species with a range of floral morphologies and staggered flowering periods, may help stabilize pollination networks in these forests. Continued investigation of nectar dynamics, pollination mechanics, competitive interactions, and plant recruitment will be necessary to evaluate the long‐term persistence of these mutualisms.

More broadly, our results show that mutualistic function can persist in simplified communities through multiple behavioral pathways linking visitation, nectar dynamics, and plant reproduction. In Hawaiʻi, where extinctions and introductions have reshaped bird assemblages, our results suggest that evaluating the persistence of pollination requires tracking species visitation to flowers, mechanistic interactions with floral structures, impacts to floral rewards, and pollen‐mediated gene flow among plants. Nectar robbing was common and altered subsequent nectar production in plant‐specific ways, demonstrating that antagonistic interactions can modify floral resource availability even when fruit and seed set remain high. Quantifying these behavioral effects on nectar dynamics can improve predictions of interaction stability and inform restoration strategies that support plant recruitment and sustained pollination processes.

## Author Contributions

**Samuel B. Case:** conceptualization (lead), data curation (lead), formal analysis (lead), funding acquisition (lead), investigation (lead), methodology (lead), project administration (lead), resources (lead), supervision (lead), validation (lead), visualization (lead), writing – original draft (lead), writing – review and editing (lead). **Donald R. Drake:** conceptualization (supporting), investigation (supporting), methodology (supporting), resources (supporting), writing – review and editing (supporting). **Kevin Epperly:** investigation (supporting), methodology (supporting), resources (supporting), software (supporting), supervision (supporting), visualization (supporting). **Christopher Steinbronn:** methodology (supporting), software (supporting), visualization (supporting), writing – review and editing (supporting). **Keoki Kanakaokai:** investigation (supporting), methodology (supporting), resources (supporting). **Molly E. Hagemann:** methodology (supporting), resources (supporting), writing – review and editing (supporting). **Nathaniel H. Kingsley:** investigation (supporting), methodology (supporting), resources (supporting), writing – review and editing (supporting). **Shannon Bay Gregory:** investigation (supporting), methodology (supporting), resources (supporting). **Hanna L. Mounce:** resources (supporting), writing – review and editing (supporting). **Alejandro Rico‐Guevara:** conceptualization (supporting), funding acquisition (supporting), investigation (supporting), methodology (supporting), project administration (supporting), resources (supporting), software (supporting), supervision (supporting), writing – review and editing (supporting).

## Funding

Funding was provided by a National Science Foundation Postdoctoral Research Fellowship in Biology (NSF PRFB Grant No. 2305728) awarded to Samuel B. Case, with additional support from the Burke Museum.

## Conflicts of Interest

The authors declare no conflicts of interest.

## Supporting information


**Table S1:** Mean nectar volume (μL) and sugar concentration (% sucrose equivalents) measured before (Day 1) and 24 h after (Day 2) experimental treatments simulating nectar robbing. Treatments included control handling (Control), corolla splitting (Splitting), and corolla piercing (Piercing). Nectar volume was measured using 20 μL glass microcapillary tubes; nectar column length was measured with digital calipers and converted to volume based on tube calibration (6.4 cm = 20 μL). When nectar volume exceeded the capacity of a single tube, nectar was drawn into multiple tubes and column lengths were summed prior to conversion. Sugar concentration was measured using a handheld refractometer. Values are presented descriptively and were not included in statistical analyses because repeated nectar extraction and the 24‐h sampling interval were not designed to isolate treatment effects on nectar chemistry.
**Table S2:** Mean bill and floral dimensions used for descriptive comparisons of bird–flower trait matching. Bill length was measured as exposed culmen length (distance from the feather line at the base of the upper mandible to the bill tip) from museum specimens. Floral length was measured in the field as corolla tube length, defined as the distance from the base of the corolla tube at the ovary–corolla junction to the distal tip of the staminal column. Values represent mean ± SE; sample sizes are shown for each species.
**Table S3:** Descriptive trait‐matching indices between nectarivorous birds and lobelioid flowers calculated as the ratio of mean bill length to mean corolla tube length (bill length ÷ flower length) using values reported in Table S2. Values closer to 1 indicate closer proportional matching between bill and floral dimensions, whereas lower values indicate increasing mismatch. These metrics are provided for qualitative comparison only and were not included as predictors in statistical analyses.

## Data Availability

Data supporting this study have been uploaded as Supporting Information for peer review. Upon acceptance, these data will be archived in the Dryad Digital Repository and made publicly available.
