# Supplementary material for: Mutualism and Antagonism in a Post‐Extinction Hawaiian Bird–Lobelioid Pollination System
Source: Ecol Evol. 2026 Aug 6;16(8):e74123. doi: 10.1002/ece3.74123 (PMC13446026; doi:10.1002/ece3.74123)
Supplement: Supplementary file 1 [file ECE3-16-e74123-s001.pdf]

APPENDIX S1: Supporting Information

JOURNAL: Ecology and Evolution

TITLE: Mutualism and antagonism in a post-extinction Hawaiian bird–lobelioid pollination system

AUTHORS: Samuel B. Case, Donald R. Drake, Kevin Epperly, Christopher Steinbronn, Keoki Kanakaokai, Molly E. Hagemann, Nathaniel H. Kingsley, Shannon Bay Gregory, Hanna L. Mounce, Alejandro Rico-Guevara

CORRESPONDING AUTHOR: Samuel B. Case

EMAIL: sam.case24@gmail.com

Table S1. Mean nectar volume (µL) and sugar concentration (% sucrose equivalents) measured before (Day 1) and 24 h after (Day 2) experimental treatments simulating nectar robbing. Treatments included control handling (Control), corolla splitting (Splitting), and corolla piercing (Piercing). Nectar volume was measured using 20 µL glass microcapillary tubes; nectar column length was measured with digital calipers and converted to volume based on tube calibration (6.4 cm = 20 µL). When nectar volume exceeded the capacity of a single tube, nectar was drawn into multiple tubes and column lengths were summed prior to conversion. Sugar concentration was measured using a handheld refractometer. Values are presented descriptively and were not included in statistical analyses because repeated nectar extraction and the 24-hour sampling interval were not designed to isolate treatment effects on nectar chemistry.

Species	Treatment	Day 1 nectar volume (µL)	Day 1 sucrose (%)	Day 2 nectar volume (µL)	Day 2 sucrose (%)	N flowers
Cyanea shipmanii	Control	4.6	15.5	4.7	11.2	17
	Splitting	5.1	15.2	3.6	10.4	12
	Piercing	4.4	13.2	3.4	9.1	15
Clermontia pyrularia	Control	13.1	12.2	8.9	9.5	16
	Splitting	8.7	13.8	17.1	10.6	8
	Piercing	9.3	13.6	1.7	9.0	9
Clermontia lindseyana	Control	33.9	10.2	22.1	10.5	12
	Splitting	59.3	11.2	34.1	9.8	9
	Piercing	20.1	10.3	3.7	9.3	10

Table S2. Mean bill and floral dimensions used for descriptive comparisons of bird–flower trait matching. Bill length was measured as exposed culmen length (distance from the feather line at the base of the upper mandible to the bill tip) from museum specimens. Floral length was measured in the field as corolla tube length, defined as the distance from the base of the corolla tube at the ovary–corolla junction to the distal tip of the staminal column. Values represent mean ± SE; sample sizes are shown for each species.

Species	Measurement (source)	Mean ± SE (mm)	N
‘I‘iwi	Bill length (museum)	27.4 ± 0.3	26 specimens
‘Amakihi	Bill length (museum)	14.7 ± 0.4	9 specimens
Cyanea shipmanii	Flower length (field)	44.6 ± 0.6	7 flowers
Clermontia pyrularia	Flower length (field)	48.5 ± 1.5	4 flowers
Clermontia lindseyana	Flower length (field)	65.7 ± 3.0	3 flowers

Table S3. Descriptive trait-matching indices between nectarivorous birds and lobelioid flowers calculated as the ratio of mean bill length to mean corolla tube length (bill length ÷ flower length) using values reported in Table S2. Values closer to 1 indicate closer proportional matching between bill and floral dimensions, whereas lower values indicate increasing mismatch. These metrics are provided for qualitative comparison only and were not included as predictors in statistical analyses.

Bird Species	*Cyanea shipmanii*	*Clermontia pyrularia*	*Clermontia lindseyana*
‘I‘iwi	0.61	0.56	0.42
‘Amakihi	0.33	0.30	0.22
